# Evaluating the Quality of Studies Assessing COVID-19 Vaccine Neutralizing Antibody Immunogenicity

**DOI:** 10.3390/vaccines12111238

**Published:** 2024-10-30

**Authors:** Maeva Katzmarzyk, Robert Naughton, Ioannis Sitaras, Henning Jacobsen, Melissa M. Higdon, Maria Deloria Knoll

**Affiliations:** 1 Department of Viral Immunology, Helmholtz Center for Infection Research, 38124 Braunschweig, Germany; 2 Independent Researcher, London SW16 2TS, UK; 3 W. Harry Feinstone Department of Molecular Microbiology and Immunology, Johns Hopkins Bloomberg School of Public Health, Baltimore, MD 21205, USA; 4 International Vaccine Access Center, Department of International Health, Johns Hopkins Bloomberg School of Public Health, Baltimore, MD 21205, USA

**Keywords:** SARS-CoV-2, neutralizing antibodies, COVD-19, reliability, reporting quality

## Abstract

**Objective:** COVID-19 vaccine-neutralizing antibodies provide early data on potential vaccine effectiveness, but their usefulness depends on study reliability and reporting quality. **Methods:** We systematically evaluated 50 published post-vaccination neutralizing antibody studies for key parameters that determine study and data quality regarding sample size, SARS-CoV-2 infection, vaccination regimen, sample collection period, demographic characterization, clinical characterization, experimental protocol, live virus and pseudo-virus details, assay standardization, and data reporting. Each category was scored from very high to low or unclear quality, with the lowest score determining the overall study quality score. **Results:** None of the studies attained an overall high or very high score, 8% (*n* = 4) attained moderate, 42% (*n* = 21) low, and 50% (*n* = 25) unclear. The categories with the fewest studies assessed as ≥ high quality were SARS-CoV-2 infection (42%), sample size (30%), and assay standardization (14%). Overall quality was similar over time. No association between journal impact factor and quality score was found. **Conclusions:** We found that reporting in neutralization studies is widely incomplete, limiting their usefulness for downstream analyses.

## 1. Introduction

The COVID-19 pandemic is an ongoing threat to global health. With its emergence in late 2019, the severe acute respiratory syndrome coronavirus 2 (SARS-CoV-2) spread globally, causing over 700 million COVID-19 infections and over 7 million deaths worldwide by January 2024 [1]. Vaccination campaigns, non-lethal infections, and public health strategies bolstered immunity and curtailed the virus’s spread. Continuously emerging variants, however, necessitate adaptations of authorized vaccines. Consequently, various vaccine platforms and new vaccine formulations that target new subvariants have been introduced into immunization programs and require ongoing performance evaluation. New products are authorized for use based on clinical trials assessing induced immunogenicity relative to products already in use. The clinical effectiveness of COVID-19 vaccines under current conditions can only be assessed by observational studies after they have been in widespread use. However, policy decisions and recommendations for vaccines against emerging variants are needed before robust clinical evidence becomes available. For timely decision-making, neutralizing antibody studies serve as a rapid surrogate for assessing vaccine efficacy.

Neutralizing antibody studies have been pivotal in understanding immunity induced by vaccination or infection, vaccine effectiveness, and viral susceptibility to neutralization [2,3,4,5,6]. As neutralizing antibodies have remained the principal correlate of protection against COVID-19 [7,8,9], these studies have the potential to directly inform vaccine policy. For example, data on immunogenicity played a key role in policy decisions around the introduction of booster vaccinations in the United States [10]. While neutralizing antibody studies continue to play a vitally important role in our understanding of COVID-19 vaccine performance, they are not without limitations. Although multiple types of in vitro neutralization assays have demonstrated robustness and comparability of results [11], several meta-analyses have demonstrated high heterogeneity between studies [2,12,13,14]. For example, in one meta-analysis, fold-reductions in neutralizing antibody titers against Omicron BA.1 relative to the index strain following first mRNA booster vaccination ranged from 1-fold to over 30-fold across studies [2]. The key concern in these studies lies in the lack of consistency, which results in heterogeneous study design and methods, and the execution of respective assays often varies between laboratories, limiting the comparability of study results. Methods reported often lack sufficient detail on aspects that could clarify reasons for obtaining different results. Despite the important role these studies have played in the current pandemic, to the best of our knowledge, there has been no assessment of the quality of these data with respect to comparability, robustness, and usefulness for downstream applications, like meta-analyses.

Neutralization results can be strongly affected by technical parameters that frequently vary across studies, including, but not limited to, “infectious virus input” and “time between infection and measurement” of infected cells in neutralization assays [15]. This emphasizes the importance of consistency and comprehensive reporting of details in neutralizing antibody studies. In the context of immune response and neutralization titers, the impact of non-technical parameters such as sample size, immunocompromising conditions of study participants, and time elapsed between infection/vaccination and sera collection will impact results. In an effort to reduce uncertainty in neutralization evidence to facilitate an improved understanding of vaccine performance under varying conditions, we created a Quality Assessment Tool (QAT) based on a previously published tool that includes key parameters likely affecting neutralization results [16].

The QAT enables a standardized, systematic, and objective evaluation of neutralizing antibody studies by assessing the overall quality and reliability of reported neutralization titers based on the information provided within each study. The tool assesses study characteristics, including methodological accuracy, experimental setting, characterization of cohorts, samples, and viruses, as well as completeness and quality of reporting. In addition to serving as an instrument to assess quality and enhance inter-study comparability, the QAT can serve as a checklist for the systematic conduct and reporting of neutralization studies.

To understand potential reasons for heterogeneous results across studies, we used the QAT to evaluate published manuscripts on post-vaccination neutralization response to SARS-CoV-2 variants to identify which factors responsible for limitations in comparability were most frequently observed. We then assessed whether there were improvements in study quality scores based on reporting and neutralization titer reliability over time by comparing studies published early after the COVID-19 vaccine introduction to studies published approximately two years later. The main aim of this study is to emphasize the importance of consistent conduct and reporting of neutralization studies to increase downstream utility and ensure a more effective response to infectious disease outbreaks.

## 2. Methods

### 2.1. Study Selection

Since the emergence of SARS-CoV-2 Omicron, we have continuously systematically reviewed post-vaccination neutralization studies for which methods have been previously described [13]. These studies are stored in a database consisting of around 500 studies at the start of the QAT analysis. We selected 50 peer-reviewed studies, the first 25 and last 25 published studies out of that database, with the 11th of July 2023 being the cutoff point. The first 25 studies were published between February and May 2021 [17,18,19,20,21,22,23,24,25,26,27,28,29,30,31,32,33,34,35,36,37,38,39,40,41]. The last 25 studies were published between March and July 2023 (Supplementary Table S1; [42,43,44,45,46,47,48,49,50,51,52,53,54,55,56,57,58,59,60,61,62,63,64,65,66]). Within the systematic literature review, studies that solely evaluated partial vaccination included samples solely collected from immunocompromised subjects or used variants of concern (e.g., Omicron) as the reference strain were excluded. Moreover, we excluded studies that used surrogate neutralization assays to evaluate neutralizing antibody titers and studies that reported NT80 or similar results instead of NT50. We solely included pseudo-virus studies that use reporter viruses and quantify infected cells via a luciferase fluorescent signal. Studies that report fold reductions in neutralization titers or studies that report data that enable the calculation of fold reduction in neutralization titers were included. All details of the search strategy and inclusion criteria can be derived from previous publications [2,12,13].

### 2.2. Quality Assessment Tool (QAT) Development and Update

We updated the previously published tool to incorporate booster vaccinations and breakthrough infections and to optimize additional parameters for clarity in the application [16] (Table 1; https://view-hub.org/sites/default/files/2024-09/QAT%20checklist%20view-hub_20240909.pdf (accessed on 28 October 2024)). Briefly, this tool employs 34 key questions, referred to as “parameters”, linked to eleven categories: sample size, SARS-CoV-2 infection, vaccination regimen, sample collection period, demographic characterization, clinical characterization, protocol, live virus, pseudo-virus, assay standardization, and data reporting. The parameters were identified through our experience in the design, conduct, and analysis of studies on neutralizing antibodies, in reviewing the literature on SARS-CoV-2 neutralization, and from discussions and collaboration with experts in this field. Answers to the parameters aim to determine the study’s quality in each category depending on the perceived extent of their effect on the comparability, reliability, and robustness of the reported neutralization titers, which is then translated into a qualitative assessment from “low” to “very high” quality. Briefly, each parameter is attributed to a specific response that carries an impact on the quality in the respective category (Supplementary Table S2). For example, a large sample size increases the quality, while a small sample size reduces quality. Some parameters with stratifications can have more complex outcomes. For example, the outcomes for the parameter “Was presence or absence of pre-vaccination infection confirmed?” are “Yes”, “No/ Not reported”, and “Not applicable”. Whereas outcomes for the parameter “Was any SARS-CoV-2 infection prior to completion of the primary vaccine regimen considered?” are “Yes—only naïve included”, “Yes—participants stratified by prior infection status”, “Reported but not considered”, and “Not reported”. When parameters are “not applicable” to the study, they have no impact on the quality.

An “unclear” rating was assigned when reporting for one or more parameters was insufficient to evaluate the quality based on that parameter. Unclear ratings contribute to the assessment of reporting quality, which we defined as completeness of information and was distinguished from the reliability assessment. In this context, under-reporting and missing details are indicators of low reporting quality. The overall quality of the study was determined by the lowest score obtained across all categories (“Very high” > “High” > “Moderate” > “Unclear” > “Low”). To regulate the impact of the quality scores of certain categories on the overall score, each category is attributed the maximum possible impact. The categories of sample size, SARS-CoV-2 infection, sample collection period, demographic and clinical characterization, and assay standardization can obtain the lowest possible quality score, “Low”, whereas vaccination regimen, protocol, live and pseudo-virus details, and data reporting can obtain an “Unclear” score as the lowest score as they have a lesser impact on the study quality. Due to this assessment being qualitative and based on provided data from the literature, we did not quantify the exact impact or uncertainty resulting from parameters with unclear or low-quality scores.

### 2.3. QAT Evaluation Process

To ensure unbiased evaluations, each study was randomly assigned to two of four experienced evaluators, who are authors of this manuscript, and independently assessed each study. Scoring discrepancies of paired assessments were discussed and resolved by the two assigned evaluators. Each study was evaluated based on the details provided within the publication.

The QAT evaluation process was managed using REDCap electronic data capture tools hosted at Johns Hopkins University [67,68].

### 2.4. Data Analysis

Within each category, we calculated the distribution of quality scores across studies. We compared the percentage of studies with very high or high scores vs. the remaining scores and the percentage with unclear quality ratings vs. the remaining scores for each category between studies published in 2021 and 2023 using chi-square or Fisher’s exact tests; the Wilcoxon rank-sum test was used to compare medians. The Student’s t-test was performed to assess differences in the average proportion of scores across studies between those published in 2021 vs. 2023. A Spearman correlation was used to assess the correlation between the studies’ overall quality scores and the impact factor of the journal the study was published in. Generation of graphs, tables, and statistical calculations were performed using Excel 2016 and SAS software 9.4.

## 3. Results

### 3.1. Evaluation of Reliability and Reporting Quality Across COVID-19 Studies

**Figure 1:**
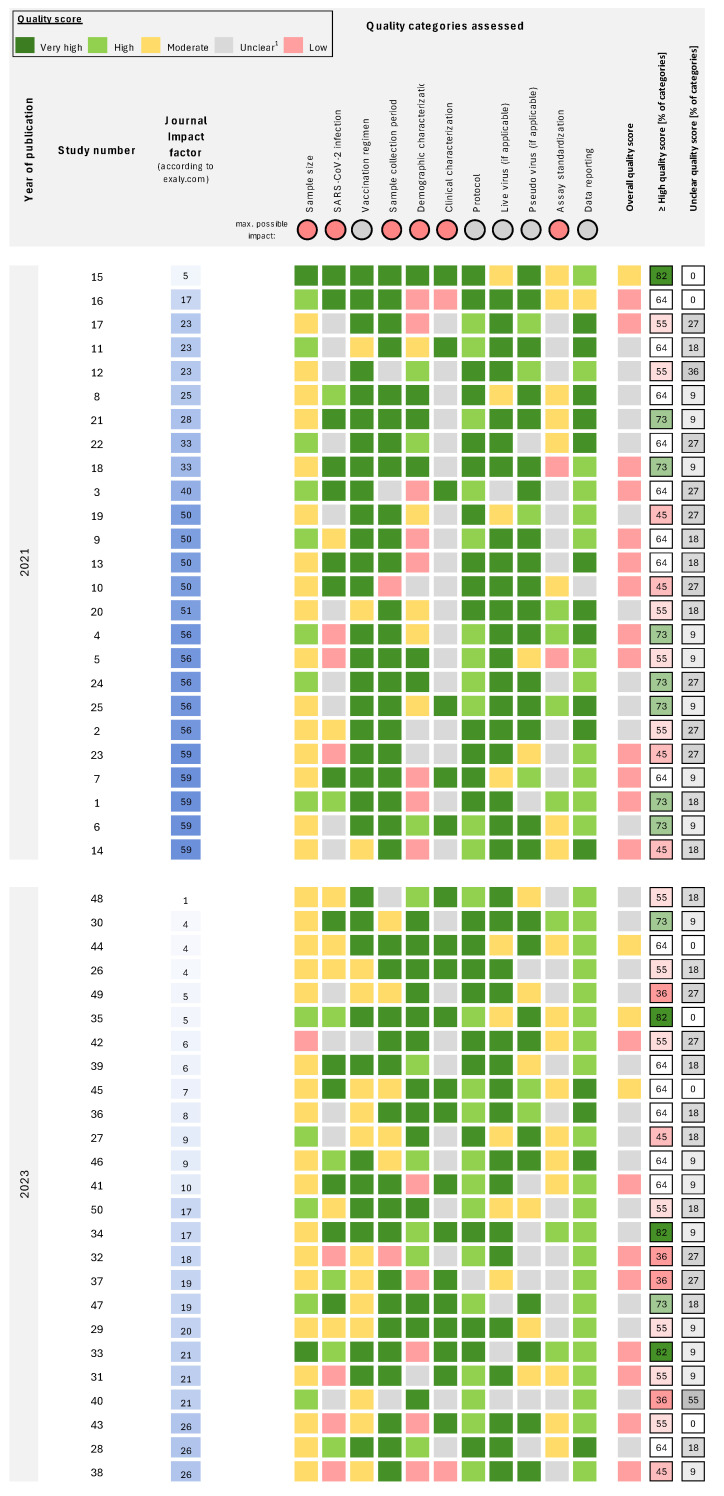
**Individual quality scores for each category of all assessed studies. Studies are sorted for impact factor and publication year.** References [17,18,19,20,21,22,23,24,25,26,27,28,29,30,31,32,33,34,35,36,37,38,39,40,41,42,43,44,45,46,47,48,49,50,51,52,53,54,55,56,57,58,59,60,61,62,63,64,65,66] to the respective study number is provided in Supplementary Table S1. Quality scores from low to very high are provided for eleven categories. The maximum impact is “unclear” for the categories of vaccination regimen, protocol, live virus, pseudo-virus, and data reporting; all remaining categories have “low” as the lowest quality score. Overall quality is assigned the lowest score observed across categories. For each study, the percentage of the 11 categories with high or very high quality scores and unclear scores is provided. ^1^ Unclear quality is specified as no sufficient information to evaluate the quality of the data, indicating low reporting quality.

**Figure 2:**
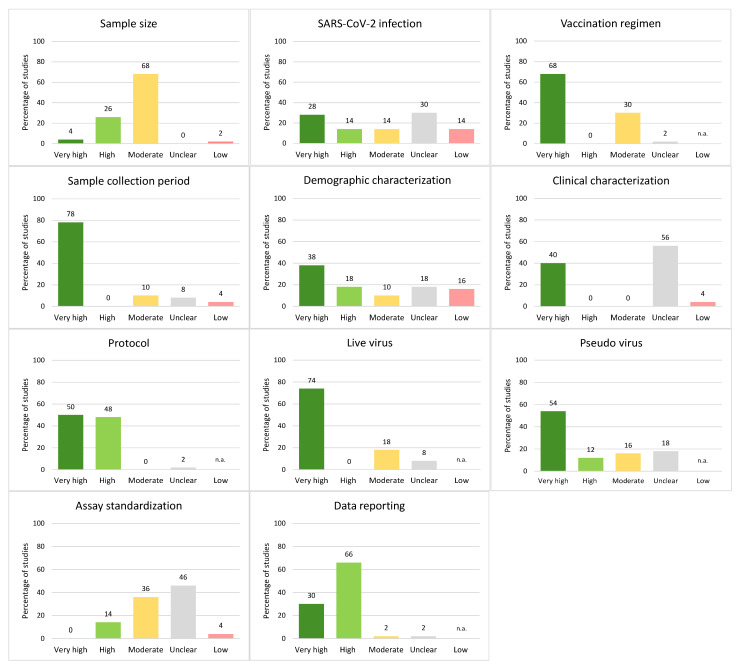
**Percentage of studies receiving specific quality scores for each category of the QAT.** Quality ratings from low to very high quality scores on the x-axis. If the maximum possible impact of a category is unclear, low quality is not applicable (n.a.) for that category. The percentage of studies with the respective quality scores is provided above the bars.

Almost all studies (44/50, 88%) had at least one category with unclear quality, and no study attained a high or very high overall quality score: 8% (*n* = 4) had moderate overall quality, 42% (*n* = 21) low and 50% (*n* = 25) unclear (Figure 1). The median percentage of categories with high or very high quality scores was 63.6% (*IQR*: 54.6–70.5%). Categories with the highest percentage of studies assigned a high or very high quality score included protocol (98%), data reporting (96%), sample collection period (78%), live virus details (74%), and vaccination regimen (68%; Figure 2); categories with the fewest studies assessed as very high or high quality were SARS-CoV-2 infection (42%), sample size (30%) and assay standardization (14%).

The median percentage of categories with an unclear quality score was 18.2% (*IQR*: 9.1–27.3%; Figure 1). Categories with the highest proportion of studies with unclear reporting were clinical characterization (56%), assay standardization (46%), and SARS-CoV-2 infection (30%; Figure 2). The most common issues were a lack of reporting of the health status of study participants (see parameters in the category “clinical characterization”), as well as underreporting of details on the neutralization assay, which includes reporting of the actual virus input in the assay and the use of a confirmation method if any. In addition, minor details on cell culture were frequently missing. Moreover, information on infections prior to vaccinations or breakthrough infections was often not considered. All studies reported sample size and sample collection period.

### 3.2. Temporal Trends in Reporting Quality and Reliability of COVID-19 Studies

**Figure 3:**
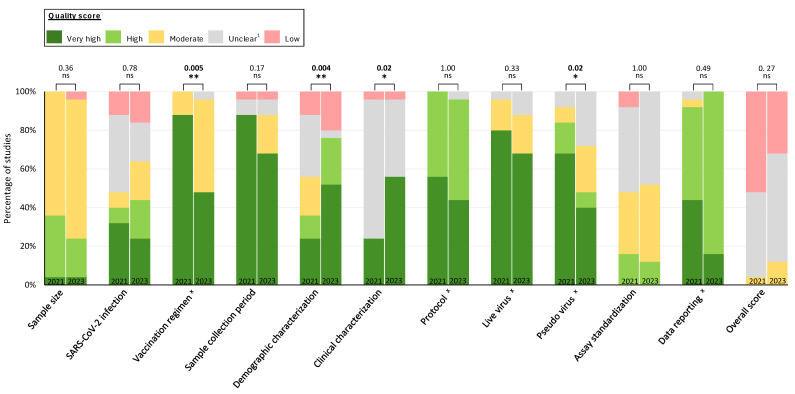
**Comparison of quality scores for each category of studies published in 2021 vs. 2023.** The percentage of studies with very high, high, moderate, unclear, and low quality scores are shown by publication year (February–May 2021 and March–July 2023) for 25 studies in each period. *p*-values for comparisons of very high or high vs. other categories are provided for each category. ^x^ The maximum impact is “unclear” for the categories of vaccination regimen, protocol, live virus, pseudo-virus, and data reporting; all remaining categories have “low” as the maximum quality score. Overall quality is assigned the lowest score observed across categories. ^1^ Unclear quality is specified as no sufficient information to evaluate the quality of the data, indicating low reporting quality. Statistical analysis was performed by Student’s t-test. Statistical significance was defined by a value of * <0.05; ** <0.01; ns, not significant.

Overall quality scores were similar between studies published in 2021 compared to 2023, with 4% (*n* = 1), 52% (*n* = 13), and 44% (*n* = 11) attaining moderate, low, and unclear quality scores, respectively, in 2021 compared to 12% (*n* = 3), 32% (*n* = 8), and 56% (*n* = 14) in 2023 (Figure 3). The median percentage of categories with a high or very high quality score (63.6% (*IQR*: 54.6–72.7%) vs. 54.5% (*IQR*: 54.6–63.6%), *p* = 0.26) or an unclear score (18.2% (*IQR*: 9.1–27.3%) vs. 18.2% (*IQR*: 9.1–18.2%), *p* = 0.25; calculated from Figure 1) was similar between 2021 and 2023 studies.

Quality scores improved between 2021 and 2023 for demographic characterization (36% vs. 76% high or very high quality, *p* = 0.004) and clinical characterization (24% vs. 56%, *p* = 0.02), as did the proportion with an unclear rating for these categories (demographic characterization: 32% vs. 4%, *p* = 0.02; clinical characterization: 72% vs. 40%, *p* = 0.02; Supplementary Figure S1). However, quality scores declined for vaccination regimen (88% vs. 48%, *p* = 0.005) and pseudo-virus (84% vs. 48%, *p* = 0.002; Figure 3). No statistically significant differences between periods were observed for other categories.

The 2021 studies were published in journals with a higher impact factor than the 2023 studies (median 50 (*IQR*: 28–56.2) vs. 10 (5.6–19.7), *p* < 0.0001). However, no meaningful correlation was found between the impact factor and the median percentage of categories with a high or very high rating (Spearman correlation [r_s_] = −0.01, *p* = 0.94) or the median percentage of categories with unclear scores (r_s_ = 0.19, *p* = 0.18).

## 4. Discussion

Systematic application of a tool assessing quality as data reliability and reporting quality of studies evaluating SARS-CoV-2 antibody neutralization in response to COVID-19 vaccination identified notable limitations in published reports. Many of these limitations, which could be easily addressed, contribute to uncertainty surrounding heterogeneous findings and thus limit the usefulness of these studies. No study received an overall assessment of high or very high quality scores, but most studies provided precise details on the neutralization assay protocol, reported and confirmed the virus lineage of live virus strains used in the assay, used reasonable reference virus strains for calculating variant-specific fold-changes, and provided appropriate statistics and/or raw neutralization data. In addition, the majority of studies assessed here considered general factors affecting immune response in the study cohort, such as age, infecting variants from pre-vaccination or from breakthrough infections, and comparable sample collection periods after vaccination or infection.

We observed the lowest quality scores in the assay standardization category, which suggests low consideration of the impact of assay details on resulting neutralizing antibody titers. For example, reporting and confirmation of infectious virus input in the assay were predominantly neglected (see Supplementary Figure S2), yet previous studies have shown that these small details can greatly affect assay results [15,69]. In addition, since neutralization assays are cell-based, it is vital to report details on cell culture, including confluence, culture conditions, and, most importantly, maximum passage number. Quality in terms of reliability would also be improved by applying a higher standard for the assay details, including considering and confirming comparable infectious virus inputs by performing back titrations or using virus input controls and allowing an appropriate variance of titers.

When assessing reporting quality, we observed a general lack of information critical for evaluating quality across all assessed studies. Reporting of details on the study cohort, including prior infection history and health status, as well as details on the assay system used, which can strongly influence study results, were overlooked. Details like the health status of study participants, sequence confirmation of used (pseudo-) virus, virus input, and cell culture details, whilst known in respective labs, are often underestimated in their significance. Incorporating this information would enhance the study’s value for data comprehension.

When comparing studies from 2021 and 2023, we found no consistent improvement in reliability and reporting quality over time, as improvements in some categories were offset by declines in others. The lack of improvement could be partly attributed to sampling bias, which resulted in the inclusion of fewer studies with rigorous methods later in the pandemic. Reporting quality and reliability in the categories of demographic and clinical characterization improved from 2021 to 2023 despite the more complex epidemiological landscape in 2023, which was marked by diverse vaccine regimens and increased infection-induced immunity in populations. A greater experience with these neutralization studies over time might have improved the overall detail of reporting in these categories. In addition, in 2023, infection-derived immunity was more common and likely resulted in a greater emphasis on cohort descriptions. We recognize the critical role of reporting pre-vaccination infections, breakthrough infections, and infecting strains in the current intricate immunological landscape as essential for understanding resulting neutralizing antibody titers. Moreover, accurate demographic and clinical characterizations of the study cohort play a pivotal role in assessing the potential impact of age, sex, or health status on the immune response and, by extension, on neutralizing antibody titers within cohorts.

In contrast, studies published in 2021 provided greater details on vaccination regimens and pseudo-virus details than 2023 publications, probably because the first COVID-19 vaccines had recently started to circulate and felt more important to distinguish between different vaccines. Moreover, the parameters in the vaccination regimen category mainly include details on reporting and comparability of booster dosing intervals within the study cohort. As booster vaccinations were not common in 2021 compared to 2023, these parameters were not applicable and hence resulted in no reduction of quality in these studies, which could be an additional explanation for the differences between 2021 and 2023. Studies published later in the pandemic show frequent underreporting of details on the pseudo-virus system. Since it is known that single amino acid mutations in the spike of RNA viruses can alter the immune escape [15,70,71,72,73], reporting and sequence confirmation of spike mutations included in the pseudo-virus spike is crucial to evaluate the comparability of resulting neutralizing antibody titers. Recently published studies often referred to previous publications for details on the pseudo-virus system, which often did not provide the relevant information. Since pseudo-virus strains are typically obtained from reliable sources and virus alterations are unlikely, our tool rates the potential underreporting or lower data reliability in the pseudo-virus category as less influential.

Cochrane is a global network known for producing high-quality, evidence-based health research, including tools and frameworks for clinical trials and observational studies. Their Risk of Bias 2 (RoB 2) and Risk of Bias in Non-randomized Studies of Interventions (ROBINS-I) tools are widely used in systematic reviews and meta-analyses to assess the quality of individual studies [74,75]. These tools could be utilized to evaluate the bias in sample selection, measurement of bias, and reporting of bias from neutralizing antibody studies. However, they lack parameters like the variability in reagents (e.g., virus strains), differences in experimental setups (incubation times, cell lines for different assays), and technical issues that are highly relevant for neutralizing antibody studies. We consider a tool tailored to studies on neutralizing antibodies a valuable contribution to the field.

One limitation of this study is the lack of a gold standard against which to measure the validity of the QAT. While we believe the tool provides an objective, qualitative assessment of study quality with a focus on reliability and reporting quality, it should be used discerningly. Based on research findings, a group of experts discussed and agreed upon the parameters included in the QAT to impact neutralization results. However, a “low quality score” does not necessarily imply biased results; rather, it ensures that comparability between studies is limited and differences due to other factors cannot be ruled out. A more refined weighting of each parameter and category by its ability to influence results could optimize the tool further, but the benefit of the tool simply to guide conduct and reporting of neutralization studies details could broaden their usefulness. Another limitation of the study is the relatively small number of studies used for assessment; a larger number of studies conducted across diverse periods and settings would enhance representativeness. However, despite the small numbers, this review was sufficient to identify characteristics in studies in both early and recent time periods that could be improved.

This study provided insights that can guide the conduct and reporting of future neutralization studies, emphasizing the importance of consistent practices that can contribute to a more effective response to the COVID-19 pandemic and future infectious disease outbreaks. Neutralizing antibodies remain the primary correlate of protection against COVID-19, providing rapid insights into the neutralization escape of novel emerging SARS-CoV-2 variants or the performance of vaccine candidates. While individual studies may provide high-quality information, their power to inform policy is limited, which usually requires corroboration among several independent studies. Meta-analyses increase the robustness of evidence, but only when studies have high comparability and reliability, highlighting the importance of their systematic conduct and reporting. The application of checklists can enhance consistency, improve study reliability, and elucidate sources of heterogeneity between studies, thereby elevating the utility of such studies across various applications, particularly in informing policy. The substantial underreporting across various categories that this assessment identified, especially in cohort details and assay-related aspects, indicates a lack of consistent protocols and limited emphasis on reporting quality across laboratories and by reviewers for scientific journals. Through comprehensive and consistent reporting across laboratories, we can enhance the reliability and utility of neutralization studies and advance the quality of research in this critical area.

## Figures and Tables

**Table 1:** Parameters of each category assessed in the QAT.

Categories		Parameters	
1	Sample size	1.1	How many samples were included?
2	SARS-CoV-2 infection	2.1	Was any SARS-CoV-2 infection prior to completion of the primary vaccine regimen considered?
		2.2	Was presence or absence of pre-vaccination infection confirmed?
		2.3	Were breakthrough infections considered in the study cohort?
		2.4	Were breakthrough infections confirmed?
		2.5	Were infection-naïve/previously infected/ breakthrough infected samples stratified in the analyses?
3	Vaccination regimen	3.1	Do the authors report booster dosing interval?
		3.2	Are the booster dosing intervals comparable?
		3.3	Do the authors stratify for partial and complete primary regimen?
4	Sample collection period	4.1	Were all samples taken at least seven days post last immunogenic event?
		4.2	Are the results stratified, OR are all samples taken ≥ two weeks and ≤ 4 months post-last immunogenic event?
5	Demographic characterization	5.1	Is the age distribution of all subjects reported?
		5.2	Are results stratified by age group?
		5.3	Is the sex distribution of all participants reported?
		5.4	If only a subgroup of the initial study cohort was analyzed, did the cohort selection happen unbiased?
		5.5	Was the infecting variant/ or variant prevalence reported?
		5.6	Was the study period and geographic location reported?
		5.7	If (multiple) breakthrough infections occurred, were the results stratified for the infecting variant(s)?
6	Clinical characterization	6.1	Is any relevant clinical characterization reported?
		6.2	Are the results stratified for immunocompromised?
7	Protocol	7.1	Is the precise assay type and endpoint reported?
		7.2	Do the authors provide a precise protocol for the neutralization assay within the manuscript?
8	Live virus	8.1	Is the virus lineage reported?
		8.2	Has the sequence been confirmed by sequencing?
9	Pseudo-virus	9.1	Are the construct details reported?
		9.2	Are all variant-associated spike mutations included in the pseudo-virus?
		9.3	Has the sequence been confirmed by sequencing?
10	Assay standardization	10.1	Is the amount of infectious virus input (virus titer) used for neutralization assays reported, and if so, are they consistent and have a small input variance?
		10.2	Was the intended virus titer used for neutralization assays confirmed by back-titration or virus controls?
		10.3	Are precise details on cell culture reported?
11	Data reporting	11.1	Is the raw data for neutralization titers reported?
		11.2	Is the reference virus used for calculating variant-specific fold changes reasonable?
		11.3	Are appropriate statistics provided?

## Data Availability

The original contributions presented in the study are included in the article/Supplementary Materials. Further inquiries can be directed to the corresponding authors.

## Supplementary Materials

The following supporting information can be downloaded at: https://www.mdpi.com/article/10.3390/vaccines12111238/s1, Table S1: Table including all 50 included neutralizing antibody studies published in 2021 and 2023, Table S2: Significance of each parameter present in the QAT is described. All possible outcomes and their respective impact on the quality score of each parameter is presented in the right columns; File S1: ASSAY DETAILS.
