# Supplementary material for: Evaluating the Quality of Studies Assessing COVID-19 Vaccine Neutralizing Antibody Immunogenicity
Source: Vaccines (Basel). 2024 Oct 30;12(11):1238. doi: 10.3390/vaccines12111238 (PMC11598362; doi:10.3390/vaccines12111238)
Supplement: Supplementary file 1 [file vaccines-12-01238-s001.zip › vaccines-3239185-supplementary.pdf]

Evaluating the quality of studies assessing COVID-19 vaccine neutralizing antibody immunogenicity

Supplementary data

Maeva Katzmarzyk[1], Robert Naughton[2], Ioannis Sitaras[3], Henning Jacobsen[1], Melissa M. Higdon[4*], and Maria Deloria Knoll[4]

[1] Department of Viral Immunology, Helmholtz Center for Infection Research, 38124, Braunschweig, Germany; maeva.katzmarzyk@helmholtz-hzi.de; henning.jacobsen@helmholtz-hzi.de

[2] Independent Researcher , SW16 2TS, London, UK; robertnaughton98@gmail.com

[3] W. Harry Feinstone Department of Molecular Microbiology and Immunology, Johns Hopkins Bloom-berg School of Public Health, Baltimore, MD 21205, USA; ioannis_sitaras@hotmail.com

[4] International Vaccine Access Center, Department of International Health, Johns Hopkins Bloomberg School of Public Health, Baltimore, MD 21205, USA; mknoll2@jhu.edu

* Correspondence: mhigdon@jhu.edu

Supplementary table 1: Table including all 50 included neutralizing antibody studies published in 2021 and 2023.

Study Number	Reference	Publication
1	17	Xie, X., Liu, Y., Liu, J., Zhang, X., Zou, J., Fontes-Garfias, C. R., ... & Shi, P. Y. (2021). Neutralization of SARS-CoV-2 spike 69/70 deletion, E484K and N501Y variants by BNT162b2 vaccine-elicited sera. Nature medicine, 27(4), 620-621.
2	18	Zhou, D., Dejnirattisai, W., Supasa, P., Liu, C., Mentzer, A. J., Ginn, H. M., ... & Screaton, G. R. (2021). Evidence of escape of SARS-CoV-2 variant B. 1.351 from natural and vaccine-induced sera. Cell, 184(9), 2348-2361.
3	19	Garcia-Beltran, W. F., Lam, E. C., Denis, K. S., Nitido, A. D., Garcia, Z. H., Hauser, B. M., ... & Balazs, A. B. (2021). Multiple SARS-CoV-2 variants escape neutralization by vaccine-induced humoral immunity. Cell, 184(9), 2372-2383.
4	20	Wang, P., Casner, R. G., Nair, M. S., Wang, M., Yu, J., Cerutti, G., ... & Ho, D. D. (2021). Increased resistance of SARS-CoV-2 variant P. 1 to antibody neutralization. Cell host & microbe, 29(5), 747-751.
5	21	Collier, D. A., De Marco, A., Ferreira, I. A., Meng, B., Datir, R. P., Walls, A. C., ... & Visualization Johnson Rob 79. (2021). Sensitivity of SARS-CoV-2 B. 1.1. 7 to mRNA vaccine-elicited antibodies. Nature, 593(7857), 136-141.
6	22	Muik, A., Wallisch, A. K., Sänger, B., Swanson, K. A., Mühl, J., Chen, W., ... & Şahin, U. (2021). Neutralization of SARS-CoV-2 lineage B. 1.1. 7 pseudovirus by BNT162b2 vaccine–elicited human sera. Science, 371(6534), 1152-1153.
7	23	Wu, K., Werner, A. P., Koch, M., Choi, A., Narayanan, E., Stewart-Jones, G. B., ... & Edwards, D. K. (2021). Serum neutralizing activity elicited by mRNA-1273 vaccine. New England Journal of Medicine, 384(15), 1468-1470.
8	24	Madhi, S. A., Baillie, V., Cutland, C. L., Voysey, M., Koen, A. L., Fairlie, L., ... & Izu, A. (2021). Efficacy of the ChAdOx1 nCoV-19 Covid-19 vaccine against the B. 1.351 variant. New England Journal of Medicine, 384(20), 1885-1898.
9	25	Hoffmann, M., Arora, P., Groß, R., Seidel, A., Hörnich, B. F., Hahn, A. S., ... & Pöhlmann, S. (2021). SARS-CoV-2 variants B. 1.351 and P. 1 escape from neutralizing antibodies. Cell, 184(9), 2384-2393.
10	26	Kuzmina, A., Khalaila, Y., Voloshin, O., Keren-Naus, A., Boehm-Cohen, L., Raviv, Y., ... & Taube, R. (2021). SARS-CoV-2 spike variants exhibit differential infectivity and neutralization resistance to convalescent or post-vaccination sera. Cell host & microbe, 29(4), 522-528.
11	27	Edara, V. V., Norwood, C., Floyd, K., Lai, L., Davis-Gardner, M. E., Hudson, W. H., ... & Suthar, M. S. (2021). Infection-and vaccine-induced antibody binding and neutralization of the B. 1.351 SARS-CoV-2 variant. Cell host & microbe, 29(4), 516-521.
12	28	Stamatatos, L., Czartoski, J., Wan, Y. H., Homad, L. J., Rubin, V., Glantz, H., ... & McGuire, A. T. (2021). mRNA vaccination boosts cross-variant neutralizing antibodies elicited by SARS-CoV-2 infection. Science, 372(6549), 1413-1418.
13	29	Planas, D., Bruel, T., Grzelak, L., Guivel-Benhassine, F., Staropoli, I., Porrot, F., ... & Schwartz, O. (2021). Sensitivity of infectious SARS-CoV-2 B. 1.1. 7 and B. 1.351 variants to neutralizing antibodies. Nature medicine, 27(5), 917-924.
14	30	Emary, K. R., Golubchik, T., Aley, P. K., Ariani, C. V., Angus, B., Bibi, S., ... & Pollard, A. J. (2021). Efficacy of ChAdOx1 nCoV-19 (AZD1222) vaccine against SARS-CoV-2 variant of concern 202012/01 (B. 1.1. 7): an exploratory analysis of a randomised controlled trial. The Lancet, 397(10282), 1351-1362.
15	31	Dejnirattisai, W., Zhou, D., Supasa, P., Liu, C., Mentzer, A. J., Ginn, H. M., ... & Screaton, G. R. (2021). Antibody evasion by the P. 1 strain of SARS-CoV-2. Cell, 184(11), 2939-2954.
16	32	Shen, X., Tang, H., McDanal, C., Wagh, K., Fischer, W., Theiler, J., ... & Montefiori, D. C. (2021). SARS-CoV-2 variant B. 1.1. 7 is susceptible to neutralizing antibodies elicited by ancestral spike vaccines. Cell host & microbe, 29(4), 529-539.
17	33	Wang, P., Nair, M. S., Liu, L., Iketani, S., Luo, Y., Guo, Y., ... & Ho, D. D. (2021). Antibody resistance of SARS-CoV-2 variants B. 1.351 and B. 1.1. 7. Nature, 593(7857), 130-135.
18	34	Liu, Y., Liu, J., Xia, H., Zhang, X., Fontes-Garfias, C. R., Swanson, K. A., ... & Shi, P. Y. (2021). Neutralizing activity of BNT162b2-elicited serum. New England Journal of Medicine, 384(15), 1466-1468.
19	35	Liu, Y., Liu, J., Xia, H., Zhang, X., Zou, J., Fontes-Garfias, C. R., ... & Shi, P. Y. (2021). BNT162b2-elicited neutralization against new SARS-CoV-2 spike variants. New England Journal of Medicine, 385(5), 472-474.
20	36	Bates, T. A., Leier, H. C., Lyski, Z. L., McBride, S. K., Coulter, F. J., Weinstein, J. B., ... & Tafesse, F. G. (2021). Neutralization of SARS-CoV-2 variants by convalescent and BNT162b2 vaccinated serum. Nature communications, 12(1), 5135.

21	37	Wang, G. L., Wang, Z. Y., Duan, L. J., Meng, Q. C., Jiang, M. D., Cao, J., ... & Ma, M. J. (2021). Susceptibility of circulating SARS-CoV-2 variants to neutralization. New England Journal of Medicine, 384(24), 2354-2356.
22	38	Geers, D., Shamier, M. C., Bogers, S., den Hartog, G., Gommers, L., Nieuwkoop, N. N., ... & GeurtsvanKessel, C. H. (2021). SARS-CoV-2 variants of concern partially escape humoral but not T cell responses in COVID-19 convalescent donors and vaccine recipients. Science immunology, 6(59), eabj1750.
23	39	Anichini, G., Terrosi, C., Gori Savellini, G., Gandolfo, C., Franchi, F., & Cusi, M. G. (2021). Neutralizing antibody response of vaccinees to SARS-CoV-2 variants. Vaccines, 9(5), 517.
24	40	Cao, Y., Yisimayi, A., Bai, Y., Huang, W., Li, X., Zhang, Z., ... & Xie, X. S. (2021). Humoral immune response to circulating SARS-CoV-2 variants elicited by inactivated and RBD-subunit vaccines. Cell research, 31(7), 732-741.
25	41	Becker, M., Dulovic, A., Junker, D., Ruetalo, N., Kaiser, P. D., Pinilla, Y. T., ... & Schneiderhan-Marra, N. (2021). Immune response to SARS-CoV-2 variants of concern in vaccinated individuals. Nature communications, 12(1), 3109.
26	42	Tan, N. H., Geers, D., Sablerolles, R. S., Rietdijk, W. J., Goorhuis, A., Postma, D. F., ... & van der Kuy, P. H. M. (2023). Immunogenicity of bivalent omicron (BA. 1) booster vaccination after different priming regimens in health-care workers in the Netherlands (SWITCH ON): results from the direct boost group of an open-label, multicentre, randomised controlled trial. The Lancet Infectious Diseases, 23(8), 901-913.
27	43	Chen, J. J., Li, L. B., Peng, H. H., Tian, S., Ji, B., Shi, C., ... & Wang, G. L. (2023). Neutralization against XBB. 1 and XBB. 1.5 after omicron subvariants breakthrough infection or reinfection. The Lancet Regional Health–Western Pacific, 33.
28	44	Chen, X., Ciric, C., Gibson, T., Anderson, L. J., Anderson, E. J., & Rostad, C. A. (2023, April). Longitudinal Neutralizing and Functional Antibody Responses to Severe Acute Respiratory Syndrome Coronavirus 2 Variants Following Messenger RNA Coronavirus Disease 2019 Vaccination. In Open Forum Infectious Diseases (Vol. 10, No. 4, p. ofad167). US: Oxford University Press.
29	45	Chen, Z., Huang, T., He, T., Zha, G., Zhu, Q., Zhang, G., ... & Ren, H. (2023). Humoral responses after primary and booster SARS-CoV-2 inactivated vaccination in patients with chronic hepatitis B virus infection: A longitudinal observational study. Journal of Medical Virology, 95(4), e28695.
30	46	Yamasoba, D., Uriu, K., Plianchaisuk, A., Kosugi, Y., Pan, L., Zahradnik, J., ... & Sato, K. (2023). Virological characteristics of the SARS-CoV-2 omicron XBB. 1.16 variant. The Lancet Infectious Diseases, 23(6), 655-656.
31	47	Ren, W., Zhang, Y., Rao, J., Wang, Z., Lan, J., Liu, K., ... & Ding, Q. (2023). Evolution of immune evasion and host range expansion by the SARS-CoV-2 B. 1.1. 529 (Omicron) variant. MBio, 14(2), e00416-23.
32	48	Guo, L., Zhang, Q., Zhong, J., Chen, L., Jiang, W., Huang, T., ... & Wang, J. (2023). Omicron BA. 1 breakthrough infections in inactivated COVID-19 vaccine recipients induced distinct pattern of antibody and T cell responses to different Omicron sublineages. Emerging Microbes & Infections, 12(1), 2202263.
33	49	Liu, Y., Wang, Z., Zhuang, X., Zhang, S., Chen, Z., Zou, Y., ... & Cheng, G. (2023). Inactivated vaccine-elicited potent antibodies can broadly neutralize SARS-CoV-2 circulating variants. Nature Communications, 14(1), 2179.
34	50	Hernández, J., Dehesa-Canseco, F., Vázquez-López, A. B., Reséndiz-Sandoval, M., Caire-Juvera, G., Solís-Hernández, M., ... & Mata-Haro, V. (2023). Neutralization of Omicron BA. 1, BA. 5.1. 6, BQ. 1.3 and XBB1. 1 induced by heterologous vaccination Ad5-nCoV and mRNA-1273. Signal Transduction and Targeted Therapy, 8(1), 174.
35	51	Diem, G., Jäger, M., Dichtl, S., Bauer, A., Lass-Flörl, C., Reindl, M., ... & Posch, W. (2023). Vaccination and Omicron BA. 1/BA. 2 Convalescence Enhance Systemic but Not Mucosal Immunity against BA. 4/5. Microbiology Spectrum, 11(3), e05163-22.
36	52	Yamamoto, S., Matsuda, K., Maeda, K., Oshiro, Y., Inamura, N., Mizoue, T., ... & Ohmagari, N. (2023). Omicron BA. 1 neutralizing antibody response following Delta breakthrough infection compared with booster vaccination of BNT162b2. BMC Infectious Diseases, 23(1), 282.
37	53	Cui, Z., Luo, W., Chen, R., Li, Y., Wang, Z., Liu, Y., ... & Li, W. (2023). Comparing T-and B-cell responses to COVID-19 vaccines across varied immune backgrounds. Signal Transduction and Targeted Therapy, 8(1), 179.
38	54	Faraone, J. N., Qu, P., Evans, J. P., Zheng, Y. M., Carlin, C., Anghelina, M., ... & Liu, S. L. (2023). Neutralization escape of Omicron XBB, BR. 2, and BA. 2.3. 20 subvariants. Cell Reports Medicine, 4(5).
39	55	Carr, E. J., Wu, M. Y., Gahir, J., Harvey, R., Townsley, H., Bailey, C., ... & Wall, E. C. (2023). Neutralising immunity to omicron sublineages BQ. 1.1, XBB, and XBB. 1.5 in healthy adults is boosted by bivalent BA. 1-containing mRNA vaccination and previous Omicron infection. The Lancet Infectious Diseases, 23(7), 781-784.
40	56	Wang, W., Goguet, E., Paz, S., Vassell, R., Pollett, S., Mitre, E., & Weiss, C. D. (2023). Bivalent coronavirus disease 2019 vaccine antibody responses to Omicron variants suggest that responses to divergent variants would be improved with matched vaccine antigens. The Journal of Infectious Diseases, 228(4), 439-443.
41	57	Vikse, E. L., Fossum, E., Erdal, M. S., Hungnes, O., & Bragstad, K. (2023). Poor neutralizing antibody responses against SARS-CoV-2 Omicron BQ. 1.1 and XBB in Norway in October 2022. Influenza and Other Respiratory Viruses, 17(6), e13144.

42	58	Toyoda, M., Tan, T. S., Motozono, C., Barabona, G., Yonekawa, A., Shimono, N., ... & Ueno, T. (2023). Evaluation of Neutralizing Activity against Omicron Subvariants in BA. 5 Breakthrough Infection and Three-Dose Vaccination Using a Novel Chemiluminescence-Based, Virus-Mediated Cytopathic Assay. Microbiology spectrum, 11(4), e00660-23.
43	59	Yang, J., Hong, W., Lei, H., He, C., Lei, W., Zhou, Y., ... & Wei, X. (2023). Low levels of neutralizing antibodies against XBB Omicron subvariants after BA. 5 infection. Signal Transduction and Targeted Therapy, 8(1), 252.
44	60	Muangnoicharoen, S., Wiangcharoen, R., Nanthapisal, S., Kamolratakul, S., Lawpoolsri, S., Jongkaewwattana, A., ... & Pitisuttithum, P. (2023). Single Ad26. COV2. S booster dose following two doses of BBIBP-CorV vaccine against SARS-CoV-2 infection in adults: Day 28 results of a phase 1/2 open-label trial. Vaccine, 41(32), 4648-4657.
45	61	Radion, E. I., Mukhin, V. E., Kholodova, A. V., Vladimirov, I. S., Alsaeva, D. Y., Zhdanova, A. S., ... & Yudin, S. M. (2023). Functional characteristics of serum Anti-SARS-CoV-2 antibodies against Delta and Omicron variants after vaccination with Sputnik V. Viruses, 15(6), 1349.
46	62	Jin, P. F., Guo, X. L., Gou, J. B., Hou, L. H., Song, Z. Z., Zhu, T., ... & Zhu, F. C. (2023). Immunogenicity and safety of heterologous immunisation with Ad5-nCOV in healthy adults aged 60 years and older primed with an inactivated SARS-CoV-2 vaccine (CoronaVac): a phase 4, randomised, observer-blind, non-inferiority trial. The Lancet Regional Health–Western Pacific, 38.
47	63	Dowell, A. C., Lancaster, T., Bruton, R., Ireland, G., Bentley, C., Sylla, P., ... & Moss, P. (2023). Immunological imprinting of humoral immunity to SARS-CoV-2 in children. Nature Communications, 14(1), 3845.
48	64	Varasi, I., Lai, A., Fiaschi, L., Bergna, A., Gatti, A., Caimi, B., ... & Vicenti, I. (2023). Neutralizing antibodies response to novel SARS-CoV-2 omicron sublineages in long-term care facility residents after the fourth dose of monovalent BNT162b2 COVID-19 vaccination. Journal of Infection, 87(3), 270-272.
49	65	Wang, F., Huang, B., Deng, Y., Zhang, S., Liu, X., Wang, L., ... & Tan, W. (2023). Neutralizing antibody levels associated with injectable and aerosolized Ad5-nCoV boosters and BA. 2 infection. BMC medicine, 21(1), 233.
50	66	Lyke, K. E., Atmar, R. L., Dominguez Islas, C., Posavad, C. M., Deming, M. E., Branche, A. R., ... & Roberts, P. C. (2023). Immunogenicity of NVX-CoV2373 heterologous boost against SARS-CoV-2 variants. npj Vaccines, 8(1), 98.

Supplementary table 2: Significance of each parameter present in the QAT is described. All possible outcomes and their respective impact on the quality score of each parameter is presented in the right columns.

COHORT DETAILS		
**1 SAMPLE SIZE**		
**1.1** How many samples were included?	Outcomes	Quality score
Required to assess the statistical strength, potential for spurious results and overall generalizability of results. Because probability of spurious results can vary with technical quality of the study and degree of heterogeneity of the population from which specimens were selected with respect to confounding factors, a single threshold cannot be identified, but larger N can mitigate these influences.<br>The reviewer should consider population heterogeneity and technical study quality to adjust the threshold and/or quality score for each study.	>50	VERY HIGH
	21-50	HIGH
	5-20	MODERATE
	<5	LOW
	Not reported	UNCLEAR
**2 SARS-COV-2 INFECTION**		
**2.1** Was any SARS-CoV-2 infection prior to completion of the primary vaccine regimen considered?	Outcomes	Quality score
There is accumulating evidence that convalescent subjects develop a stronger immune response to vaccination compared to SARS-CoV-2 naïve subjects (Rössler et al., 2023; Saadat et al., 2021; Stamatatos et al., 2021; Usdan et al., 2024; Vicenti et al., 2021). Therefore, it is important to provide sufficient information about pre-vaccination COVID-19 in the study population to consider this aspect as a potential risk of bias.<br>If no information on this aspect is available, the respective reliability of data is unclear, affecting study quality. If only naïve participants are included, or results stratified by pre-vaccination infection status, no risk of bias can be assumed, and therefore data quality can be expected to be very high. When pre-vaccination infection is reported, but not considered in the results, we recommend assuming a high risk of bias and low quality for this criterion if ≥20% of the study cohort were infected prior to vaccination.	Yes - only naïve included	VERY HIGH
	Yes – participants stratified by prior infection status	VERY HIGH
	Reported but not considered	LOW
	Not reported	UNCLEAR
**2.2** Was presence or absence of pre-vaccination infection confirmed?	Outcomes	Quality score
Self-report is likely to have low sensitivity since not everyone would be tested, and asymptomatic infections may not trigger testing. Additionally, self-reported negative test results may not reflect status at the time of specimen collection. Because the potential impact of non-naïve subjects is high, the study population should be screened by the investigators for pre-vaccination COVID-19 by sensitive methods (e.g., NP-ELISA or by repeated qPCR screening or antigen testing over the whole study period (and pre-study period if applicable). If the pre-vaccination COVID-19 status is reported but not confirmed, we assign a quality score of high, because there is still a risk of unreported/ unconfirmed cases although it is likely, that most cases are considered by the respective screening-method.	Yes	VERY HIGH
	No / not reported	HIGH
	N. a.	VERY HIGH
**2.3** Were breakthrough infections considered in the study cohort?	Outcomes	Quality score
Especially in longitudinal studies, breakthrough cases of COVID-19 might appear in the study cohort. Like pre-vaccination infections, these infections are known to affect the subject's immune response and neutralization titers because of boosting-like effects. If breakthrough cases are likely to appear in the study setting (e.g., longitudinal studies), this can pose a significant bias. Hence, we assign a moderate quality score for not considering breakthrough cases in the study cohort.	Yes	VERY HIGH
	No	MODERATE
	N. a.	VERY HIGH

**2.4** Were breakthrough infections confirmed?	Outcomes	Quality score
Like mentioned for the pre-vaccination infections, presence of absence of breakthrough infections should be screened and confirmed by investigators. For a previously infection-naïve study population, this can be done by performing assessments of longitudinal clinical and/ or diagnostic follow-up or subject-specific antibody responses.	Yes	VERY HIGH
	No	HIGH
	N. a.	VERY HIGH

**2.5** Were infection-naïve/previously infected/ breakthrough infected samples stratified in the analyses?	Outcomes	Quality score
If breakthrough cases of COVID-19 are reported for the study cohort, neutralization results should be stratified for naïve and infected subjects to acknowledge booster-effects of the infection. We recommend applying this criterion if for ≥20% of the study cohort breakthrough cases are reported. Missing stratification can result in a high risk of bias, dependent on the number of affected subjects.	Yes	VERY HIGH
	No	LOW
	N. a.	VERY HIGH

3 VACCINATION REGIMEN

**3.1** Do the authors report booster dosing interval?	Outcomes	Quality score
There is increasing evidence that the dosing interval for vaccines with a prime-boost regimen can affect the immune response including neutralization titers (Guo et al., 2023). We therefore recommend considering the dosing interval in interpretation of the data and assign missing information a moderate quality score.	Yes	VERY HIGH
	No	MODERATE
	N. a.	VERY HIGH

**3.2** Are the booster dosing intervals comparable?	Outcomes	Quality score
Booster dosing intervals on the contrary to the primary vaccinations are not harmonized within the population. Shorter or longer dosing intervals were shown, however, to affect neutralizing antibody titers (Guo et al., 2023). Hence, when comparing neutralizing antibody titers within and across cohorts, we assign a moderate quality score if the intervals are within a four-month range, and if < 20% of samples do not comply. Missing information on the dosing interval is assigned an unclear quality score, indicating low reporting quality.	Yes	VERY HIGH
	No	MODERATE
	Not reported	UNCLEAR
	N.a.	VERY HIGH

**3.3** Do the authors stratify for partial and complete primary regimen?	Outcomes	Quality score
Certain studies investigate neutralization titers from individuals with partial and full primary vaccine regimen. It is imperative that these cohorts are completely separated, as it is known that titers post first primary vaccination and post full primary regimen are significantly different (Behrens et al., 2022). We assign a moderate quality score for this aspect because most studies only include small numbers of partially immunized subjects into the respective study. Studies involving greater proportions of non-stratified subjects (> 20%) should not be considered.	Yes	VERY HIGH
	No	MODERATE
	N. a.	VERY HIGH

4 SAMPLE COLLECTION PERIOD

**4.1** Were all samples taken at least seven days post last immunogenic event?	Outcomes	Quality score
Because of the kinetics of neutralizing antibody generation, no samples taken ≤ 7 days post last immunogenic event (infection or vaccination) should be considered (Collier Ai-ris Y. et al., 2021; Doria-Rose Nicole et al., 2021; Levin Einav G. et al., 2021). We recommend excluding any study involving ≥20 % of subjects with samples taken earlier than 7 days post last immunogenic event from further consideration.	Yes	VERY HIGH
	No	LOW
	Not reported	UNCLEAR

**4.2** Are the results stratified OR are all samples taken ≥ two weeks and ≤ 4 months post last immunogenic event?	Outcomes	Quality score
Peak neutralization titers are usually observed 14-28 days post last immunogenic event followed by a gradual decline of neutralization activity (waning) (Collier Ai-ris Y. et al., 2021; Doria-Rose Nicole et al., 2021; Levin Einav G. et al., 2021). When assessing neutralization results and especially when comparing studies, it is important to acknowledge these kinetics by stratification of the results <u>or</u> by only including subjects sampled within a range of peak titers. Based on currently available literature, we defined 4 months post last dose as the upper limit for this period (Jacobsen et al., 2023). Since the literature shows gradual declines in neutralizing antibodies within the first six months of up to 5-fold, we assign a moderate quality score when this aspect is not considered or reported. We recommend applying this criterion if for ≥20% of the study cohort stratification or proper study design is missing.	Yes	VERY HIGH
	No	MODERATE
	Not reported	MODERATE
	N. a.	VERY HIGH

5 DEMOGRAPHIC CHARACTERIZATION

**5.1** Is the age distribution of all subjects reported?	Outcomes	Quality score
As for many other pathogens, age is highly likely to also affect neutralization titers against SARS-CoV-2, especially when overall low responses are reported (Bates et al., 2021; Collier et al., 2021; Müller et al., 2021). Therefore, the age structure of the study cohort should be reported to allow proper interpretation of results. Because the effect of age on anti-SARS-CoV-2 neutralization is not yet fully understood, but was shown to affect neutralizing antibodies, we assign a moderate quality score for this aspect (Bates et al., 2022; Holtkamp et al., 2023; Vanetti et al., 2023).	Yes	VERY HIGH
	No	MODERATE

**5.2** Are results stratified by age group?	Outcomes	Quality score
To acknowledge effects of age on neutralization titers, we recommend stratifying results based on age groups, especially for older adults (≥ 60 years), adults and children (< 18 years), if ≥20 % of the study cohort belong to different age groups. We still assign a high quality score for missing age-stratification if the age distribution is not reported, because context-specific interpretation remains possible.	Yes	VERY HIGH
	No	HIGH
	Not reported	HIGH

**5.3** Is the sex distribution of all participants reported?	Outcomes	Quality score
Although there is conflicting data on the effect of the biological sex on neutralization titers against SARS-CoV-2 (Tsverava et al., 2022), more recent studies suggest that biological sex does not significantly affect neutralizing antibody data (Yan et al., 2022; Yang et al., 2023; H. Zhang et al., 2022; X. Zhang et al., 2023). Since detailed reporting of the study cohort, including characteristics like sex, implies good scientific practice and reporting quality, we assign a high quality score when these details about the study cohort are not reported.	Yes	VERY HIGH
	No	HIGH

**5.4** If only a subgroup of the initial study cohort was analyzed, did the cohort selection happen unbiased?	Outcomes	Quality score
In most studies, total IgG-titers are assessed along with neutralization titers. Some studies chose to then separate the study cohort into separate groups of "responders" based on the antibody response for further analysis. If neutralization titers are assessed, it is essential that no biased pre-selection was performed on the study cohort. Results restricted to, for example elite-responders or non-responders should not be considered for routine use and analysis.	Yes	VERY HIGH
	No	LOW
	N.a.	VERY HIGH
	Not reported	UNCLEAR

**5.5** Was the infecting variant/ or variant prevalence reported?	Outcomes	Quality score
If post-vaccination (breakthrough) SARS-CoV-2 infections occurred during the study, it appears important to consider these events for the assessment of the neutralization response. For this, it is important to understand which SARS-CoV-2 variants caused infection, because there is increasing evidence, that variants can have differential effects on the neutralization response. By now we know that variant-specific infections highly affect the neutralizing antibody titers depending on the tested variant (Faraone et al., 2023; Yisimayi et al., 2024). Hence, we assign a low quality score if the infecting variant is not reported. If the infecting variant is not provided, the prevalence of variants at the time of infection can alternatively help to understand and correctly interpret data in the context of SARS-CoV-2 infections that occurred during or before the study period. This question is only applicable if SARS-CoV-2 breakthrough infections occurred.	Yes	VERY HIGH
	No	LOW
	N. a.	VERY HIGH

**5.6** Was the study period and geographic location reported?	Outcomes	Quality score
As mentioned above. The infecting variant is important to understand resulting neutralizing antibody titers. Hence, if the infecting variant and the prevalence at the time of infection is not provided within the manuscript, the study period and geographic location allows predicting a distribution of variants. Because the effect of variant-specific infection on anti-SARS-CoV-2 neutralization is currently poorly understood, we assign a low risk of bias for this aspect.	Yes	VERY HIGH
	No	LOW
	N. a.	VERY HIGH

**5.7** If (multiple) breakthrough infections occurred, were the results stratified for the infecting variant(s)?	Outcomes	Quality score
As described above, the prevalence of variants can help to understand and to correctly interpret data in the context of SARS-CoV-2 infections that occurred during or before the study period. Only applicable if SARS-CoV-2 infections occurred. Because the effect of variant-specific infection on anti-SARS-CoV-2 neutralization is currently poorly understood, we assign a high quality score if the results are not stratified for infecting variants.	Yes	VERY HIGH
	No	HIGH
	N. a.	VERY HIGH

6 CLINICAL CHARACTERIZATION

**6.1** Is any relevant clinical characterization reported?	Outcomes	Quality score
Many studies assess neutralization titers in groups of individuals that are likely to have clinical characteristics that might affect the post-vaccination immune response. Some examples are immuno-suppression (more likely in older adults), frailty (more likely in women), or pregnancy (women of reproductive age only). If the study cohort might consist of a relevant proportion (≥20%) of subjects that are likely to show immune-alternating clinical characteristics, the relevant clinical characteristics of the study cohort must be reported. If this information is missing, it is not possible to assess a related risk of bias, and we recommend exclusion of this study from further analysis.	Yes	VERY HIGH
	No	UNCLEAR

**6.2** Are the results stratified for immunocompromised?	Outcomes	Quality score
If a clinical characterization is reported, we highly recommend stratifying the results for immunocompromised subjects as they might significantly affect the overall neutralization titers in a cohort (Galmiche et al., 2022; Haidar et al., 2022). Because of this, failure of stratification leads to a low quality score, yet we suggest that this aspect should only apply if ≥20% of the study cohort are eligible for stratification.	Yes	VERY HIGH
	No	LOW
	Not reported	UNCLEAR
	N. a.	VERY HIGH

ASSAY DETAILS		
**7 PROTOCOL**		
**7.1** Is the precise assay type and endpoint reported?	Outcomes	Quality score
To correctly interpret and compare studies, it is imperative that the assay type (live virus neutralization, pseudo virus neutralization; TCID, plaque-reduction neutralization etc.) along with the determined endpoint (NT20, NT50, NT80 etc.) is reported. There is by now increasing evidence that both the assay type as well as the endpoint can affect the neutralization titer (Riepler et al., 2021; Sekirov et al., 2021; von Rhein et al., 2021). If this information is missing, it is not possible to assess a related risk of bias, and we recommend exclusion of this study from further analysis.	Yes	VERY HIGH
	No	UNCLEAR
**7.2** Do the authors provide a precise protocol for the neutralization assay within the manuscript?	Outcomes	Quality score
A precise assay protocol can help to correctly interpret results and to understand differences among studies. Missing or low-quality information represent low reporting quality and reliability of data. We assign a high quality score to this aspect because most studies provide at least essential information allowing for some extent of quality assessment. However, we want to emphasize the importance of including relevant details within the manuscript, without referring to previous publications.	Yes	VERY HIGH
	No	HIGH
**8 LIVE VIRUS STRAIN (IF APPLICABLE)**		
**8.1** Is the virus lineage reported?	Outcomes	Quality score
If a live virus is used for neutralization, the lineage and origin must be reported to allow correct interpretation of results. If this information is missing, it is not possible to assess a related risk of bias, and we recommend exclusion of this study from further analysis.	Yes	VERY HIGH
	No	UNCLEAR
	N. a.	VERY HIGH
**8.2** Has the sequence been confirmed by sequencing?	Outcomes	Quality score
By now, there is substantial evidence that SARS-CoV-2 can acquire adaptational mutations in cell culture by serial passaging (Avila-Herrera et al., 2024; Chung et al., 2022). Since there is increasing evidence that mutations especially in the spike of SARS-CoV-2 can individually affect neutralization titers to a certain extent, the virus sequence should be confirmed for the passage that is used for neutralization assays (Katzmarzyk et al., 2023).<br>We assign a moderate risk of bias for this aspect when the sequence has not been confirmed by sequencing.	Yes	VERY HIGH
	No	MODERATE
	N. a.	VERY HIGH
**9 PSEUDO VIRUS STRAIN (IF APPLICABLE)**		
**9.1** Are the construct details reported?	Outcomes	Quality score
If a pseudo virus is used for neutralization, details on pseudo virus construction and origin must be reported to allow correct interpretation of results. If this information is missing, it is not possible to assess a related risk of bias, and we recommend exclusion of this study from further analysis.	Yes	VERY HIGH
	No	UNCLEAR
	N. a.	VERY HIGH
**9.2** Are all variant-associated spike mutations included to the pseudo virus?	Outcomes	Quality score
To accurately assess antibody neutralization against SARS-CoV-2 variants using a pseudo-virus system, it is important that the virus construct contains all spike-mutations that are associated to the respective variant. We recommend https://covdb.stanford.edu/ as a reference. If mutations are missing or further mutations are included in the construct, this adds a moderate quality score since several studies have shown that individual single mutations can	Yes	VERY HIGH
	No	MODERATE
	N. a.	VERY HIGH

significantly affect neutralizing antibody results using polyclonal sera (Bdeir et al., 2024; Katzmarzyk et al., 2023; Pastorio et al., 2022).		
**9.3** Has the sequence been confirmed by sequencing?	Outcomes	Quality score
To follow good scientific practice and to provide maximum credibility of the assay, we recommend confirming the pseudo virus sequence (not the plasmids) by sequencing prior to use in neutralization assays. We assign a lower quality score of "High" for missing sequencing information because pseudo-viruses are artificially generated and commonly pre-constructs such as spike-expressing plasmids are sequence confirmed.	Yes	VERY HIGH
	No	HIGH
	N. a.	VERY HIGH
**10 ASSAY STANDARDIZATION**		
**10.1** Is the amount of infectious virus input (virus titer) used for neutralization assays reported and if so, are they consistent and with small input variance?	Outcomes	Quality score
With a neutralization assay, the capability of the subject's sample (usually plasma or serum) to neutralize a defined amount of virus is measured. Standardization of input-virus is essential to provide high-quality results. The variance that is accepted for the virus input directly translates into variance of the neutralization titer. Furthermore, the quantified virus input defines sensitivity and resolution of the assay. Therefore, we consider reporting the virus titer and use of a consistent amount of virus as essential, and failure to do so as a high risk of bias and therefore, low quality score of data. If this information is missing, it is not possible to assess related data reliability and we recommend exclusion of this study from further analysis (Katzmarzyk et al., 2023; Nie et al., 2020).	Titers reported, but variance and consistency not reported	MODERATE
	Consistent and with small variance	VERY HIGH
	Not consistent or with high variance	LOW
	Not reported	UNCLEAR
**10.2** Was the intended virus titer used for neutralization assays confirmed by back-titration or virus controls?	Outcomes	Quality score
The virus input for each assay performed can be easily assessed by back titration, or by using virus input controls. This allows to precisely describe the variance conferred by the virus input and therefore optimal assessment of the assay results. Because this is mainly an aspect for quality control, we assign a high quality score if this confirmation is missing.	Yes	VERY HIGH
	No	HIGH
**10.3** Are precise details on cell culture reported?	Outcomes	Quality score
Neutralization assays are performed in a cell culture environment and the final readout is remaining infectivity of non-neutralized virus. Infectivity is highly dependent on the target cells and can be influenced by many factors such as confluency, passage number, contamination, temperature and many more. We therefore recommend reporting cell culture techniques as detailed as possible to allow optimal interpretation of results. Because this is mainly an aspect for quality control, we assign a high quality score if this confirmation is missing. We scored publications based on the following sub-questions: 1. Is the cell density reported (cell number/ confluency)? 2. Is the exact cell type reported? 3. Are the cultivation parameters (temperature, CO_2-level, medium composition) reported?	Yes	VERY HIGH
	No	HIGH

DATA		
**11 DATA REPORTING**		
**11.1** Is the raw data for neutralization titers reported?	Outcomes	Quality score
Direct reporting of raw data (ideally linked to the respective subject information such as age, sex etc.) supports optimal interpretation of results. Furthermore, raw data can be used to confirm or re-analyze statistics, if applicable. Because this is mainly an aspect for quality control, we assign a high quality score if this confirmation is missing.	Yes	VERY HIGH
	No	HIGH
**11.2** Is the reference virus used for calculating variant-specific fold-changes reasonable?	Outcomes	Quality score
In some studies, fold changes are calculated using the alpha variant as a reference. However, when using post-vaccination sera, it is important that comparisons are always made using the vaccine seed strain or a sufficiently similar strain as a reference, since the homologous comparison will determine the baseline neutralization activity of the sera, and any antigenic differences between the vaccine strain and other variants (Sitaras, 2020). Hence, we assign a moderate quality score if no reasonable reference virus was used.	Yes	VERY HIGH
	No	MODERATE
	Not reported	UNCLEAR
	N. a.	VERY HIGH
**11.3** Are appropriate statistics provided?	Outcomes	Quality score
Appropriate presentation of data and statistics can support correct interpretation of results and re-analysis as applicable. The sole presentation of for example fold-changes or bar graphs without presentation of data distribution adds uncertainty to the results and does not allow for optimal assessment. Because this is mainly an aspect for quality control, we assign a high quality score if this confirmation is missing.	Yes	VERY HIGH
	No	HIGH
	Raw data provided	VERY HIGH

Supplementary figures

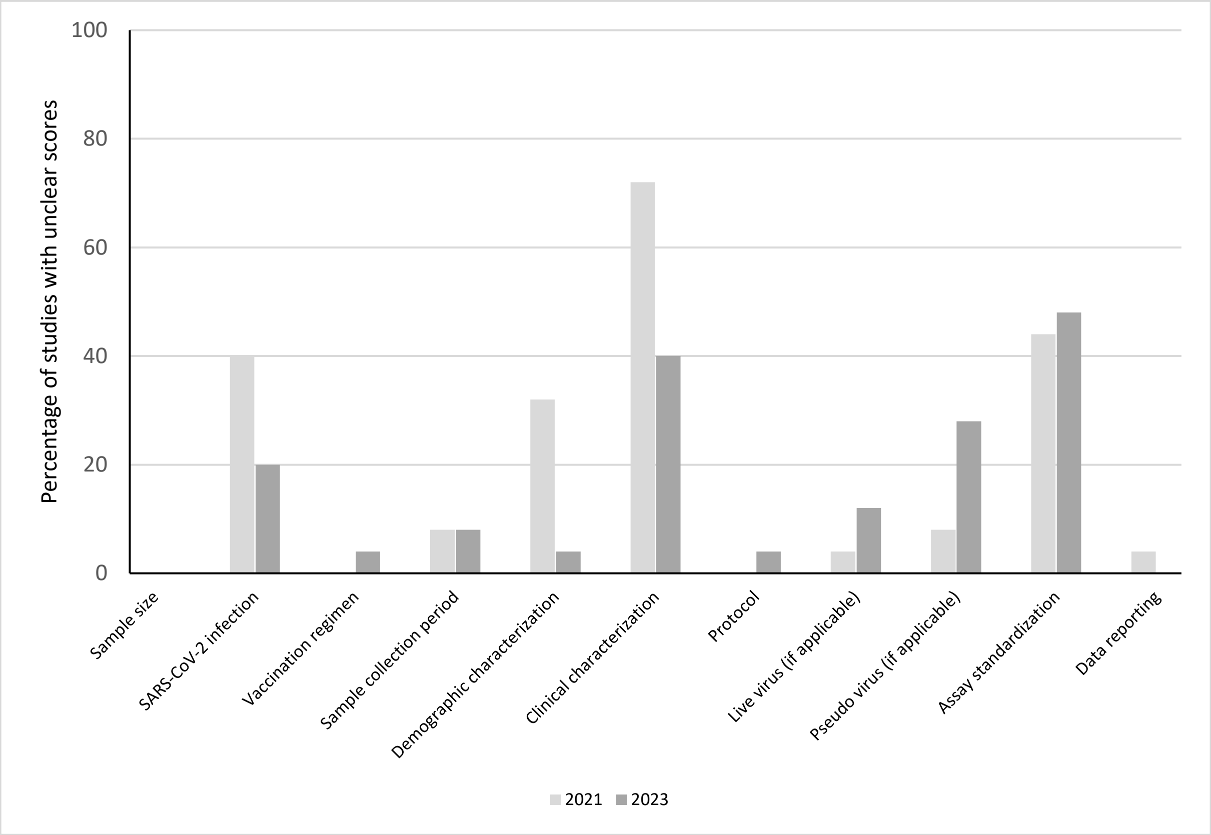

Supplementary figure 1 Percentage of studies with unclear quality scores
Percentage of studies with unclear quality scores are shown for studies in each publication year.

Supplementary figure 2 Individual quality scores for each parameter and overall quality score for the category "Assay standardization" of all assessed studies. Studies are sorted for impact factor and publication year. Quality scores from low to very high are provided

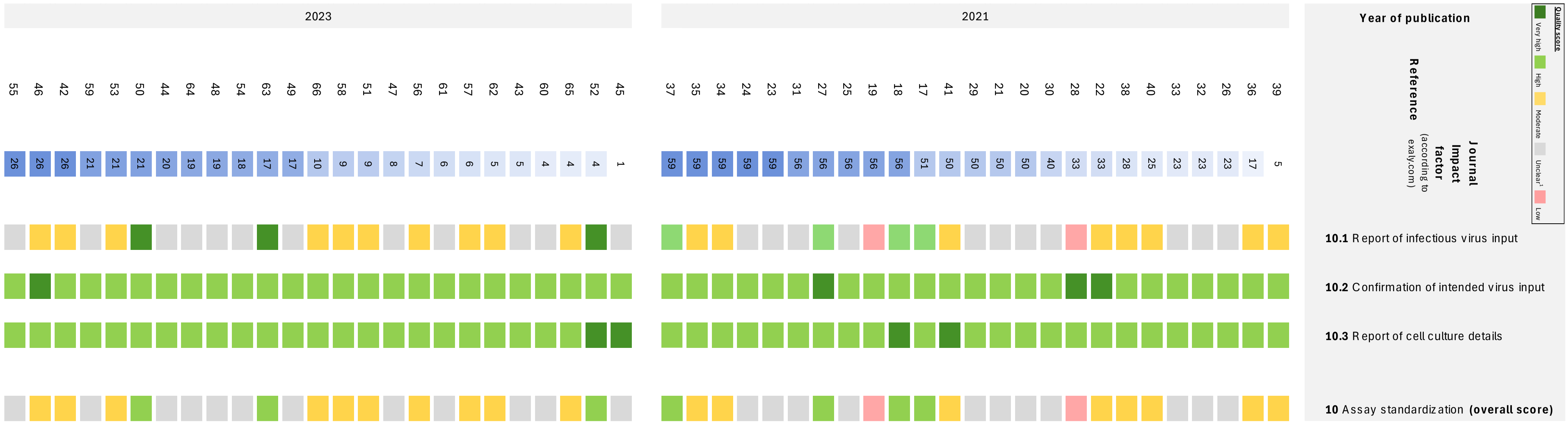

References:

Avila-Herrera, A., Kimbrel, J. A., Manuel Martí, J., Thissen, J., Saada, E. A., Weisenberger, T., Arrildt, K. T., Segelke, B. W., Allen, J. E., Zemla, A., & Borucki, M. K. (2024). Differential laboratory passaging of SARS-CoV-2 viral stocks impacts the in vitro assessment of neutralizing antibodies. *PloS One*, *19*(1), e0289198. https://doi.org/10.1371/journal.pone.0289198

Bates, T. A., Leier, H. C., Lyski, Z. L., Goodman, J. R., Curlin, M. E., Messer, W. B., & Tafesse, F. G. (2021). Age-Dependent Neutralization of SARS-CoV-2 and P.1 Variant by Vaccine Immune Serum Samples. *JAMA*, *326*(9), 868–869. https://doi.org/10.1001/jama.2021.11656

Bates, T. A., Lu, P., Kang, Y. J., Schoen, D., Thornton, M., McBride, S. K., Park, C., Kim, D., Messer, W. B., Curlin, M. E., Tafesse, F. G., & Lu, L. L. (2022). BNT162b2-induced neutralizing and non-neutralizing antibody functions against SARS-CoV-2 diminish with age. *Cell Reports*, *41*(4). https://doi.org/10.1016/j.celrep.2022.111544

Bdeir, N., Lüddecke, T., Maaß, H., Schmelz, S., Jacobsen, H., Metzdorf, K., Cossmann, A., Stankov, M. V., Hoffmann, M., Pöhlmann, S., Blankenfeldt, W., Dopfner-Jablonka, A., Behrens, G. M. N., & Čičin-Šain, L. (2024). *Reverse mutational scanning of spike BA.2.86 identifies the epitopes contributing to immune escape from polyclonal sera* (p. 2024.01.03.23300575). medRxiv. https://doi.org/10.1101/2024.01.03.23300575

Behrens, G. M. N., Barros-Martins, J., Cossmann, A., Ramos, G. M., Stankov, M. V., Odak, I., Dopfer-Jablonka, A., Hetzel, L., Köhler, M., Patzer, G., Binz, C., Ritter, C., Friedrichsen, M., Schultze-Florey, C., Ravens, I., Willenzon, S., Bubke, A., Ristenpart, J., Janssen, A., … Förster, R. (2022). BNT162b2-boosted immune responses six months after heterologous or homologous ChAdOx1nCoV-19/BNT162b2 vaccination against COVID-19. *Nature Communications*, *13*(1), 4872. https://doi.org/10.1038/s41467-022-32527-2

Chung, H., Noh, J. Y., Koo, B.-S., Hong, J. J., & Kim, H. K. (2022). SARS-CoV-2 mutations acquired during serial passage in human cell lines are consistent with several of those found in recent natural SARS-CoV-2 variants. *Computational and Structural Biotechnology Journal*, *20*, 1925–1934. https://doi.org/10.1016/j.csbj.2022.04.022

Collier Ai-ris Y., Yu Jingyou, McMahan Katherine, Liu Jinyan, Chandrashekar Abishek, Maron Jenny S., Atyeo Caroline, Martinez David R., Ansel Jessica L., Aguayo Ricardo, Rowe Marjorie, Jacob-Dolan Catherine, Sellers Daniel, Barrett Julia, Ahmad Kunza, Anioke Tochi, VanWyk Haley, Gardner Sarah, Powers Olivia, … Barouch Dan H. (2021). Differential Kinetics of Immune Responses Elicited by Covid-19 Vaccines. *New England Journal of Medicine*, *385*(21), 2010–2012. https://doi.org/10.1056/NEJMc2115596

Collier, D. A., Ferreira, I. A. T. M., Kotagiri, P., Datir, R. P., Lim, E. Y., Touizer, E., Meng, B., Abdullahi, A., Elmer, A., Kingston, N., Graves, B., Le Gresley, E., Caputo, D., Bergamaschi, L., Smith, K. G. C., Bradley, J. R., Ceron-Gutierrez, L., Cortes-Acevedo, P., Barcenas-Morales, G., … Gupta, R. K. (2021). Age-related immune response heterogeneity to SARS-CoV-2 vaccine BNT162b2. *Nature*, *596*(7872), 417–422. https://doi.org/10.1038/s41586-021-03739-1

Doria-Rose Nicole, Suthar Mehul S., Makowski Mat, O'Connell Sarah, McDermott Adrian B., Flach Britta, Ledgerwood Julie E., Mascola John R., Graham Barney S., Lin Bob C., O'Dell Sijy, Schmidt Stephen D., Widge Alicia T., Edara Venkata-Viswanadh, Anderson Evan J., Lai Lilin, Floyd Katharine, Rouphael Nadine G., Zarnitsyna Veronika, … Kunwar Pratap. (2021). Antibody Persistence through 6 Months after the Second Dose of mRNA-1273 Vaccine for Covid-19. *New England Journal of Medicine*, *384*(23), 2259–2261. https://doi.org/10.1056/NEJMc2103916

Faraone, J. N., Qu, P., Goodarzi, N., Zheng, Y.-M., Carlin, C., Saif, L. J., Oltz, E. M., Xu, K., Jones, D., Gumina, R. J., & Liu, S.-L. (2023). Immune evasion and membrane fusion of SARS-CoV-2 XBB subvariants EG.5.1 and XBB.2.3. *Emerging Microbes & Infections*, *12*(2), 2270069. https://doi.org/10.1080/22221751.2023.2270069

Galmiche, S., Nguyen, L. B. L., Tartour, E., Lamballerie, X. de, Wittkop, L., Loubet, P., & Launay, O. (2022). Immunological and clinical efficacy of COVID-19 vaccines in immunocompromised populations: A systematic review. *Clinical Microbiology and Infection*, *28*(2), 163–177. https://doi.org/10.1016/j.cmi.2021.09.036

Guo, S., Zheng, Y., Gao, Z., Duan, M., Liu, S., Du, P., Xu, X., Xu, K., Zhao, X., Chai, Y., Wang, P., Zhao, Q., Gao, G. F., & Dai, L. (2023). Dosing interval regimen shapes potency and breadth of antibody repertoire after vaccination of SARS-CoV-2 RBD protein subunit vaccine. *Cell Discovery*, *9*(1), 1–13. https://doi.org/10.1038/s41421-023-00585-5

Haidar, G., Agha, M., Bilderback, A., Lukanski, A., Linstrum, K., Troyan, R., Rothenberger, S., McMahon, D. K., Crandall, M. D., Sobolewksi, M. D., Nathan Enick, P., Jacobs, J. L., Collins, K., Klamar-Blain, C., Macatangay, B. J. C., Parikh, U. M., Heaps, A., Coughenour, L., Schwartz, M. B., … Mellors, J. W. (2022). Prospective Evaluation of Coronavirus Disease 2019 (COVID-

19) Vaccine Responses Across a Broad Spectrum of Immunocompromising Conditions: The COVID-19 Vaccination in the Immunocompromised Study (COVICS). *Clinical Infectious Diseases*, *75*(1), e630–e644. https://doi.org/10.1093/cid/ciac103

Holtkamp, C., Schöler, L., Anastasiou, O. E., Brune, B., Fessmann, K., Elsner, C., Möhlendick, B., Čiučiulkaitė, I., Dudda, M., Trilling, M., Dittmer, U., Spors, J., & Le-Trilling, V. T. K. (2023). Antibody responses elicited by mRNA vaccination in firefighters persist six months and correlate inversely with age and directly with BMI. *Heliyon*, *9*(1). https://doi.org/10.1016/j.heliyon.2022.e12746

Jacobsen, H., Sitaras, I., Katzmarzyk, M., Cobos Jiménez, V., Naughton, R., Higdon, M. M., & Deloria Knoll, M. (2023). Systematic review and meta-analysis of the factors affecting waning of post-vaccination neutralizing antibody responses against SARS-CoV-2. *Npj Vaccines*, *8*(1), 1–6. https://doi.org/10.1038/s41541-023-00756-1

Katzmarzyk, M., Clesle, D. C., van den Heuvel, J., Hoffmann, M., Garritsen, H., Pöhlmann, S., Jacobsen, H., & Čičin-Šain, L. (2023). Systematical assessment of the impact of single spike mutations of SARS-CoV-2 Omicron sub-variants on the neutralization capacity of post-vaccination sera. *Frontiers in Immunology*, *14*, 1288794. https://doi.org/10.3389/fimmu.2023.1288794

Levin Einav G., Lustig Yaniv, Cohen Carmit, Fluss Ronen, Indenbaum Victoria, Amit Sharon, Doolman Ram, Asraf Keren, Mendelson Ella, Ziv Arnona, Rubin Carmit, Freedman Laurence, Kreiss Yitshak, & Regev-Yochay Gili. (2021). Waning Immune Humoral Response to BNT162b2 Covid-19 Vaccine over 6 Months. *New England Journal of Medicine*, *385*(24), e84. https://doi.org/10.1056/NEJMoa2114583

Müller, L., Andrée, M., Moskorz, W., Drexler, I., Walotka, L., Grothmann, R., Ptok, J., Hillebrandt, J., Ritchie, A., Rabl, D., Ostermann, P. N., Robitzsch, R., Hauka, S., Walker, A., Menne, C., Grutza, R., Timm, J., Adams, O., & Schaal, H. (2021). Age-dependent Immune Response to the Biontech/Pfizer BNT162b2 Coronavirus Disease 2019 Vaccination. *Clinical Infectious Diseases*, *73*(11), 2065–2072. https://doi.org/10.1093/cid/ciab381

Nie, J., Li, Q., Wu, J., Zhao, C., Hao, H., Liu, H., Zhang, L., Nie, L., Qin, H., Wang, M., Lu, Q., Li, X., Sun, Q., Liu, J., Fan, C., Huang, W., Xu, M., & Wang, Y. (2020). Establishment and validation of a pseudovirus neutralization assay for SARS-CoV-2. *Emerging Microbes & Infections*, *9*(1), 680–686. https://doi.org/10.1080/22221751.2020.1743767

Pastorio, C., Zech, F., Noettger, S., Jung, C., Jacob, T., Sanderson, T., Sparrer, K. M. J., & Kirchhoff, F. (2022). Determinants of Spike infectivity, processing, and neutralization in SARS-CoV-2 Omicron subvariants BA.1 and BA.2. *Cell Host & Microbe*, *30*(9), 1255-1268.e5. https://doi.org/10.1016/j.chom.2022.07.006

Riepler, L., Rössler, A., Falch, A., Volland, A., Borena, W., von Laer, D., & Kimpel, J. (2021). Comparison of Four SARS-CoV-2 Neutralization Assays. *Vaccines*, *9*(1), Article 1. https://doi.org/10.3390/vaccines9010013

Rössler, A., Knabl, L., Netzl, A., Bante, D., Borena, W., von Laer, D., Smith, D. J., & Kimpel, J. (2023). Durability of Cross-Neutralizing Antibodies 5.5 Months After Bivalent Coronavirus Disease 2019 Vaccine Booster. *The Journal of Infectious Diseases*, *229*(3), 644–647. https://doi.org/10.1093/infdis/jiad472

Saadat, S., Rikhtegaran Tehrani, Z., Logue, J., Newman, M., Frieman, M. B., Harris, A. D., & Sajadi, M. M. (2021). Binding and Neutralization Antibody Titers After a Single Vaccine Dose in Health Care Workers Previously Infected With SARS-CoV-2. *JAMA*, *325*(14), 1467–1469. https://doi.org/10.1001/jama.2021.3341

Sekirov, I., Petric, M., Carruthers, E., Lawrence, D., Pidduck, T., Kustra, J., Laley, J., Lee, M.-K., Chahil, N., Mak, A., Levett, P. N., Mendoza, E., Wood, H., Drebot, M., Krajden, M., & Morshed, M. (2021). Performance comparison of micro-neutralization assays based on surrogate SARS-CoV-2 and WT SARS-CoV-2 in assessing virus-neutralizing capacity of anti-SARS-CoV-2 antibodies. *Access Microbiology*, *3*(8), 000257. https://doi.org/10.1099/acmi.0.000257

Sitaras, I. (2020). Antigenic Cartography: Overview and Current Developments. *Methods in Molecular Biology (Clifton, N.J.)*, *2123*, 61–68. https://doi.org/10.1007/978-1-0716-0346-8_5

Stamatatos, L., Czartoski, J., Wan, Y.-H., Homad, L. J., Rubin, V., Glantz, H., Neradilek, M., Seydoux, E., Jennewein, M. F., MacCamy, A. J., Feng, J., Mize, G., De Rosa, S. C., Finzi, A., Lemos, M. P., Cohen, K. W., Moodie, Z., McElrath, M. J., & McGuire, A. T. (2021). mRNA vaccination boosts cross-variant neutralizing antibodies elicited by SARS-CoV-2 infection. *Science*, *372*(6549), 1413–1418. https://doi.org/10.1126/science.abg9175

Tsverava, L., Chitadze, N., Chanturia, G., Kekelidze, M., Dzneladze, D., Imnadze, P., Gamkrelidze, A., Lagani, V., Khuchua, Z., Solomonia, R., Tsverava, L., Chitadze, N., Chanturia, G., Kekelidze, M., Dzneladze, D., Imnadze, P., Gamkrelidze, A., Lagani, V., Khuchua, Z., & Solomonia, R. (2022). Antibody profiling reveals gender differences in response to SARS-COVID-2 infection. *AIMS Allergy and Immunology*, *6*(1), Article allergy-06-01-002. https://doi.org/10.3934/Allergy.2022002

Usdan, L., Patel, S., Rodriguez, H., Xu, X., Lee, D.-Y., Finn, D., Wyper, H., Lowry, F. S., Mensa, F. J., Lu, C., Cooper, D., Koury, K., Anderson, A. S., Türeci, Ö., Şahin, U., Swanson, K. A., Gruber, W. C., Kitchin, N., & C4591044 Study Group. (2024). A Bivalent Omicron-BA.4/BA.5-Adapted BNT162b2 Booster in ≥12-Year-Olds. *Clinical Infectious Diseases: An Official Publication of the Infectious Diseases Society of America*, *78*(5), 1194–1203. https://doi.org/10.1093/cid/ciad718

Vanetti, C., Lampasona, V., Stracuzzi, M., Fenizia, C., Biasin, M., Saulle, I., Limanaqi, F., Abdelsalam, A., Loretelli, C., Paradiso, L., Longoni, E., Barcellini, L., Piemonti, L., Marzinotto, I., Dispinseri, S., Amendola, A., Fappani, C., Tanzi, E., Clerici, M. S., … Trabattoni, D. (2023). The Immunological Profile of SARS-CoV-2 Infection in Children Is Linked to Clinical Severity and Age. *International Journal of Molecular Sciences*, *24*(7), Article 7. https://doi.org/10.3390/ijms24076779

Vicenti, I., Gatti, F., Scaggiante, R., Boccuto, A., Zago, D., Basso, M., Dragoni, F., Zazzi, M., & Parisi, S. G. (2021). Single-dose BNT162b2 mRNA COVID-19 vaccine significantly boosts neutralizing antibody response in health care workers recovering from asymptomatic or mild natural SARS-CoV-2 infection. *International Journal of Infectious Diseases*, *108*, 176–178. https://doi.org/10.1016/j.ijid.2021.05.033

von Rhein, C., Scholz, T., Henss, L., Kronstein-Wiedemann, R., Schwarz, T., Rodionov, R. N., Corman, V. M., Tonn, T., & Schnierle, B. S. (2021). Comparison of potency assays to assess SARS-CoV-2 neutralizing antibody capacity in COVID-19 convalescent plasma. *Journal of Virological Methods*, *288*, 114031. https://doi.org/10.1016/j.jviromet.2020.114031

Yan, L., Liu, P., Li, X., Zhou, S., Li, H., Wang, Z., Shen, F., Lu, B., Long, Y., Xiao, X., Wang, Z., Li, D., Han, H., Yu, H., Zhou, S., Lv, W., & Yu, X. (2022). Neutralizing Antibodies and Cellular Immune Responses Against SARS-CoV-2 Sustained One and a Half Years After Natural Infection. *Frontiers in Microbiology*, *12*. https://doi.org/10.3389/fmicb.2021.803031

Yang, J., Hong, W., Lei, H., He, C., Lei, W., Zhou, Y., Zhao, T., Alu, A., Ma, X., Li, J., Yang, L., Wang, Z., Wang, W., Lu, G., Shen, G., Lu, S., Wu, G., Shi, H., & Wei, X. (2023). Low levels of neutralizing antibodies against XBB Omicron subvariants after BA.5 infection. *Signal Transduction and Targeted Therapy*, *8*(1), 1–12. https://doi.org/10.1038/s41392-023-01495-4

Yisimayi, A., Song, W., Wang, J., Jian, F., Yu, Y., Chen, X., Xu, Y., Yang, S., Niu, X., Xiao, T., Wang, J., Zhao, L., Sun, H., An, R., Zhang, N., Wang, Y., Wang, P., Yu, L., Lv, Z., … Cao, Y. (2024). Repeated Omicron exposures override ancestral SARS-CoV-2 immune imprinting. *Nature*, *625*(7993), 148–156. https://doi.org/10.1038/s41586-023-06753-7

Zhang, H., Jia, Y., Ji, Y., Cong, X., Liu, Y., Yang, R., Kong, X., Shi, Y., Zhu, L., Wang, Z., Wang, W., Fei, R., Liu, F., Lu, F., Chen, H., & Rao, H. (2022). Inactivated Vaccines Against SARS-CoV-2: Neutralizing Antibody Titers in Vaccine Recipients. *Frontiers in Microbiology*, *13*. https://doi.org/10.3389/fmicb.2022.816778

Zhang, X., Qian, C., Yang, L., Gao, H., Jiang, P., Dai, M., Wang, Y., Kang, H., Xu, Y., Hu, Q., Feng, F., Cheng, B., & Dai, E. (2023). Diagnostic value and characteristic analysis of serum nucleocapsid antigen in COVID-19 patients. *PeerJ*, *11*, e15515. https://doi.org/10.7717/peerj.15515
